# Prevalence of carbapenem-resistant Enterobacterales colonisation in hospitalised neonates

**DOI:** 10.4102/sajid.v40i1.726

**Published:** 2025-05-31

**Authors:** Michele E. Haumann, Adrie Bekker, Chandre Geldenhuys, Natasha O’Connell, Andrew Whitelaw, Tonya Esterhuizen, Angela Dramowski

**Affiliations:** 1 Department of Paediatrics and Child Health, Faculty of Medicine and Health Sciences, Stellenbosch University, Cape Town, South Africa; 2 Paediatric Department, Faculty of Medicine and Health Sciences, Karl Bremer District Hospital, Cape Town, South Africa; 3 Paediatric Department, Faculty of Medicine and Health Sciences, Khayelitsha District Hospital, Cape Town, South Africa; 4 Division of Medical Microbiology, Department of Pathology, Faculty of Medicine and Health Sciences, Stellenbosch University, Cape Town, South Africa; 5 National Health Laboratory Service, Tygerberg Hospital, Cape Town, South Africa; 6 Division of Epidemiology and Biostatistics, Department of Global Health, Faculty of Medicine and Health Sciences, Stellenbosch University, Cape Town, South Africa

**Keywords:** neonate, carbapenem resistance, rectal colonisation, preterm, prevalence

## Abstract

**Background:**

Carbapenem-resistant Enterobacterales (CRE) have emerged as major healthcare-associated infection (HAI) pathogens globally with substantial associated mortality and morbidity.

**Objectives:**

We conducted a retrospective cohort study to determine the prevalence of rectal CRE colonisation in neonates referred from a central hospital, to a regional and a district hospital in the Western Cape Province of South Africa (01 March 2019 – 30 September 2020).

**Method:**

Clinical data and laboratory records were reviewed to identify possible factors associated with CRE colonisation using stepwise forward logistic regression analysis.

**Results:**

Among 291 neonates transferred to the regional and district hospitals, the median birth weight and gestational age were 1360 (interquartile range [IQR]: 1080 g – 1690 g) and 31 (IQR: 29–33) weeks. The overall CRE rectal colonisation prevalence at the time of transfer from the central hospital was 22.3% (65/291), with colonising species including *Klebsiella pneumoniae* (59/65, 90.8%) and *Serratia marcescens* (6/65; 9.2%). There were no factors significantly associated with CRE colonisation. No CRE-colonised neonate subsequently developed CRE infection. Post-discharge mortality rates were similar in the CRE-colonised versus the non-colonised neonates (2/65 [3.1%] vs. 9/226 [4.0%]; *p* = 0.737).

**Conclusion:**

There was no increased risk of subsequent CRE infection or mortality in the 12 months post-discharge in neonates who were CRE colonised.

**Contribution:**

Rectal colonisation with CRE was highly prevalent in preterm neonates being transferred for step-down hospital care. Carbapenem-resistant Enterobacterales-colonised neonates had similar demographic characteristics to non-colonised neonates, with no factors significantly associated with CRE colonisation.

## Introduction

Neonatal bacterial infections are of global concern and account for 26% of annual neonatal deaths.^1^ The burden of neonatal infection and infection-related deaths is concentrated in low- and middle-income countries (LMICs).^1,2,3,4^

Among hospitalised neonates in South Africa, the majority of infections are healthcare-associated infection (HAI), occurring ≥ 72 h of life.^2,5,6,7,8^ Neonates in LMIC hospitals are at high risk of developing HAI and neonatal unit infection outbreaks occur frequently owing to overcrowding, sharing of equipment, and high rates of broad-spectrum antibiotic use.^9,10^

Preterm neonates who are unwell and face extended hospital stays have an increased risk of intestinal colonisation by multi-drug resistant (MDR) pathogens. This vulnerability arises from their underdeveloped immune systems and compromised skin and gastrointestinal barrier functions, making them more susceptible to invasive infections.^11,12^ The emergence of MDR gram-negative bacterial pathogens in neonatal units poses a serious public health threat and has limited options for antimicrobial treatment in LMIC neonatal units.^11,13,14^

In the last decade, carbapenem-resistant Enterobacterales (CRE) have emerged as major HAI pathogens globally.^9,11,13,15,16,17^ In general, CRE infections are associated with higher mortality rates than other non-MDR bacterial pathogens because of the limited antimicrobial treatment available and increased morbidity, such as prolonged ventilation and extended hospital stay.^5^ Highlighting the link between neonatal MDR colonisation and subsequent infection, studies have shown 21% – 50% concordance of colonisation and bloodstream infection isolates.^11,12,18,19,20^ In the last 2 years, several South African neonatal units have also experienced CRE outbreaks, which incurred high patient mortality rates and were difficult to control.^21,22^

In some high-income country neonatal units, routine screening for CRE colonisation is conducted to assist in identifying, which neonates require implementation of patient isolation and contact precautions.^23^ Although these infection prevention and control (IPC) measures have a role in limiting CRE transmission, CRE screening incurs additional costs and may have unintended negative consequences related to isolation and unnecessary antibiotics.^23^ The sensitivity of rectal swabs for CRE can vary significantly based on several factors, including the culture technique, sample storage, the specific test applied, and intermittent shedding, all of which can contribute to false negative results.^24,25,26^ Carbapenem-resistant Enterobacterales colonisation screening is seldom conducted in resource-limited neonatal units, and there are limited guidelines in South Africa to direct CRE screening. Lowman et al.’s South African Society for Clinical Microbiology (SASCM) CRE-working group (WG) consensus statement and working guidelines for the screening and laboratory detection of CRE, established in 2014, are the sole set of guidelines available in South Africa; however, they do not specifically address neonates.^25^

Similarly, there is a lack of local data on the burden of and factors associated with CRE colonisation. Given these substantial knowledge gaps, we determined the prevalence of and factors associated with rectal CRE colonisation in neonates transferred from a central hospital to a district and a regional hospital in the Western Cape Province of South Africa.

## Research methods and design

### Study setting

Tygerberg Hospital (referred to hereafter as ‘central’) is the largest hospital in Cape Town, Western Cape Province (bed capacity of 1384) and provides tertiary care to patients from the Metro East region and surrounding rural regional hospitals.^27^ The hospital’s obstetric service delivers approximately 8000 high-risk pregnancies per year, with a 37% low birth weight rate in 2018.^28^ The 132-bed neonatal unit includes a 12-bed medical and surgical neonatal intensive care unit (NICU), three 30-bed acute care wards and one 30-bed kangaroo mother care (KMC) ward. Bed occupancy rates in this central hospital neonatal unit regularly exceed 90%, necessitating regular transfer of stable, growing preterm neonates to surrounding district and regional hospitals’ neonatal units.

#### Carbapenem-resistant Enterobacterales colonisation screening protocols

In 2019 the central hospital detected the first outbreak of CRE in the neonatal unit and certain district and regional hospitals implemented a requirement to screen for CRE colonisation in neonates using rectal swabs on arrival to the down referral hospital. Before the CRE outbreak at the central hospital, no screening for CRE colonisation had been implemented. From February 2019, all neonates transferred from the central hospital to the neonatal units at Paarl Hospital (regional) and Khayelitsha District Hospital (district) had rectal swabs collected on arrival to establish their CRE colonisation status. Neonates known to be CRE colonised or infected at the central hospital were not transferred out and remained at the central hospital until discharge, further contributing to high bed occupancy rates at the central hospital. Neonates at the central hospital were not routinely screened for CRE colonisation unless they had contact with a neonate confirmed to have a CRE infection. Rectal swabs for CRE screening were taken at the district and regional hospitals on arrival and transferred to the South African National Accreditation System (SANAS) accredited National Health Laboratory Service (NHLS) microbiology laboratory at the central hospital. Most samples reached the central hospital within 24 h during weekdays and 48 h over weekends. According to the NHLS handbook, the swabs should be placed into transport media after collection and refrigerated if delays were anticipated. Because of the retrospective nature of this study, it is not possible to comment on whether all the samples were treated in the above manner, which could have affected the quality of the specimen.

At the time of the study, the laboratory protocol for screening swabs included using CARBA-SMART, a chromogenic selective medium (BioMerieux, France), for screening and then the Vitek 2 system (BioMerieux) for confirmation, identification, and susceptibility testing of isolates.

### Infection prevention and control protocols

Strict IPC protocols were enacted to avoid transmission of CRE from colonised neonates transferred to the district and regional hospitals, where there is currently no access to colistin therapy. At the regional hospital, neonates transferred from the central hospital were isolated in closed incubators and positioned at least 2 m apart in a designated isolation room. Contact precautions (gloves and aprons) were applied until a patient was confirmed to be negative on CRE rectal swab screening. Strict handwashing protocols were also enforced. Frequently touched surfaces around the patient bedside (i.e. clinical equipment, bed rails, incubator surfaces, and bedside cabinets where the patient notes are kept) were cleaned on a 12-h basis. If one of the neonates in the dedicated isolation area screened positive on their entry rectal swab, they would be moved to a separate isolation room, and all the contacts were re-swabbed and kept in isolation until their results were available.

Similar IPC precautions were used at the district hospital, although no isolation area was available. Once the CRE-colonised neonates were KMC ready (off oxygen, full feeds via cup or breastfeeding, at least 1.4 kg), they were admitted to a designated KMC room for down-referred neonates from the central hospital, where both CRE-colonised and CRE-negative neonates were kept with universal contact precautions and stringent handwashing protocols were maintained, along with surface cleaning as previously outlined, until they were ready for discharge.

### Study design

We conducted a retrospective cohort study to determine the prevalence of and factors associated with CRE rectal colonisation in neonates transferred from a central hospital to a district and a regional hospital in the Western Cape province of South Africa over 18 months (01 March 2019 – 30 September 2020). The inclusion criteria encompassed all premature and full-term neonates transferred from the central hospital, while the exclusion criteria comprised all swabs collected at district and regional hospitals that were not referred from the central facility.

Data on neonate’s CRE colonisation screening swabs was obtained from the NHLS central data warehouse. The data consisted of all CRE rectal swabs taken by both the district and regional hospitals during the 18-month period, and these were compared to the admission data on the Western Cape Provincial Department of Health Data Centre (PHDC) from both the hospital’s neonatal wards. This information was subsequently compared with medical data on electronic continuity of care record (ECCR), enterprise content management (ECM) and single patient viewer (SPV) to establish which patients met our inclusion criteria and get the information to determine if there were any factors associated with rectal CRE colonisation.

By utilising the NHLS system alongside ECCR, ECM, and SPV, all subsequent C-reactive protein levels, full blood counts with differential counts, blood cultures, urine samples, and cerebrospinal fluid (CSF) collected from the down-referred neonates were evaluated to determine if any further CRE infections had occurred.

Electronic continuity of care record, ECM, and SPV were also utilised to determine the number of patients who demised after being discharged home. In certain instances, we could identify the cause of death if available in the above-mentioned resources.

The data were captured using the Stellenbosch University Research electronic data capture (REDCap),^29,30^ a secure online electronic data capture tool hosted at Stellenbosch University.

### Data definitions

The following standard definitions were used: preterm (born before 37 weeks gestation), low birth weight (< 2500 g), very low birth weight (< 1500 g), and extremely low birth weight (< 1000 g). Neonates are typically defined as infants under 28 days of life; however, for this study, we included preterm and term infants (> 28 days of life) that remained hospitalised in a neonatal ward for weight gain. Colonisation is the presence of a microorganism on or in a host, without signs of infection. Infection is the invasion of a host organism’s bodily tissue causing disease. Carbapenem-resistant Enterobacterales are Enterobacterales that are resistant to one or more of the carbapenems.

### Data sources and aggregation

Data on neonate’s CRE colonisation screening swabs were obtained from the NHLS central data warehouse. For all neonates transferred who underwent CRE screening, clinical data were retrieved from a variety of electronic sources and then entered into the REDCap^29,30^ online data repository.

### Statistical analysis

Carbapenem-resistant Enterobacterales colonisation prevalence and hospital outcomes were described as counts and percentages with 95% confidence intervals (CIs). Normally distributed continuous data were described using means and standard deviations. Non-normally distributed data were described using medians and interquartile ranges (IQRs). Student’s *t*-test was used for comparing continuous data; the Chi-square or Fisher’s exact test was used for comparing categorical data. A *p*-value of < 0.05 was considered statistically significant. Statistical analyses were performed by using the IBM SPSS Statistics software package for Windows, Version 28.0, released 2021. Stepwise forward logistic regression analysis was performed to identify factors for CRE rectal colonisation, reporting odds ratios (ORs) and 95% CIs.

### Ethical consideration

Approval for the study (including a waiver of individual informed consent) was obtained from the Health Research Ethics Committee at Stellenbosch University (reference number: S20/11/330) and permission was obtained from the central, regional and district hospitals and Tygerberg Hospital, Paarl Hospital and Khayelitsha District Hospital management teams.

## Results

### Demographics and clinical characteristics

**FIGURE 1:**
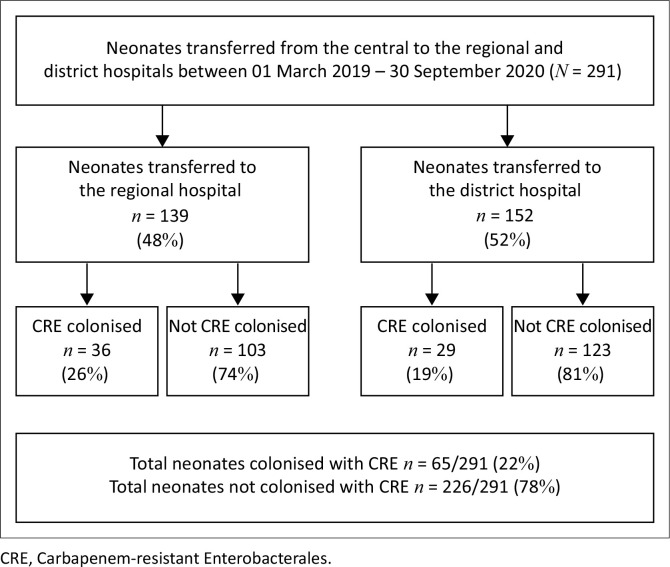
Identification of the study population.

A total of 314 rectal swabs for CRE were taken in the time period for both hospitals. Duplicate specimens and specimens from other hospitals were excluded, with a final sample of 291 (Figure 1).

Among the 291 neonates transferred to the regional and district hospitals, the median birth weight was 1360 g (IQR: 1080 g – 1690 g), and the median gestational age was 31 weeks (IQR: 29–33). A large proportion of the neonates received nasal continuous positive airway pressure (nCPAP) for respiratory distress syndrome (240/291; 82.5%) and had a long median stay at the central hospital prior to transfer of 20 days (IQR: 10–33). Very few neonates required any surgical intervention (5/291; 1.7%). Most transferred neonates were preterm (276/291; 94.8%), and half were male (145/291; 49.8%). A total of 81 neonates (28%) were exposed to HIV. Most neonates (248/291; 85%) were inborn at the central hospital (Table 1).

**TABLE 1:** Characteristics of the study population and factors associated with carbapenem-resistant Enterobacterales rectal colonisation (*N* = 291).

Characteristics	Neonates (*n* = 291)				Carbapenem-resistant Enterobacterales-colonised (*n* = 65)				Not carbapenem-resistant Enterobacterales-colonised (*n* = 226)				OR	95% CI	*p*
	*n*	%	Median	IQR	*n*	%	Median	IQR	*n*	%	Median	IQR			
**Preterm birth (< 37 weeks)**	279	95.8	-	-	63	96.9	-	-	216	95.6	-	-	-	-	0.093
< 28 weeks	22	7.6	-	-	4	6.2	-	-	18	8.0	-	-	1.444	0.229–9.106	0.695
28–31 weeks	130	44.7	-	-	37	56.9	-	-	93	41.2	-	-	2.586	0.556–12.02	0.226
32–34 weeks	105	36.1	-	-	19	29.2	-	-	86	38.1	-	-	1.950	0.402–9.459	0.407
35–37 weeks	22	7.6	-	-	3	4.6	-	-	19	8.4	-	-	0.619	0.102–3.774	0.603
**Full-term birth (> 37 weeks)**	12	4.1	-	-	2	3.1	-	-	10	4.4	-	-	-	-	-
**Birth weight (g)**	-	-	1360	1082–1690	-	-	1290	1070–1620	-	-	1400	1100–1710	-	-	0.164
ELBW	48	16.5	-	-	9	13.8	-	-	39	17.3	-	-	1.269	0.238–6.755	0.780
VLBW	129	44.3	-	-	38	58.5	-	-	91	40.3	-	-	2.279	0.486–10.85	0.294
LBW	101	34.7	-	-	16	24.6	-	-	85	37.6	-	-	1.035	0.209–5.120	0.966
> 2500 g	13	4.5	-	-	2	3.1	-	-	11	4.9	-	-	-	-	-
Male gender	145	49.8	-	-	31	47.7	-	-	114	50.4	-	-	0.898	0.516–1.556	0.696
Exposed to HIV	81	27.8	-	-	17	26.2	-	-	64	28.3	-	-	0.896	0.480–1.674	0.732
Inborn at Tygerberg Hospital	248	85.2	-	-	60	92.3	-	-	188	83.2	-	-	1.925	0.744–4.786	0.159
**Feeds on arrival:**															
Exclusive breast milk	229	78.7	-	-	53	81.5	-	-	176	77.9	-	-	0.772	0.364–1.641	0.501
Formula feeds and mixed feeding	53	18.2	-	-	10	15.4	-	-	43	19.0	-	-	0.949	0.191–4.705	0.949
Unknown	9	3.1	-	-	2	3.1	-	-	7	3.1	-	-	-	-	-
Requiring CPAP	240	82.5	-	-	52	80.0	-	-	188	83.2	-	-	0.809	0.401–1.629	0.552
**Surfactant given**	-	-	-	-	-	-	-	-	-	-	-	-	0.886	0.438–1.794	0.737
1 dose	40	13.7	-	-	7	10.8	-	-	33	14.6	-	-	-	-	0.689
2 doses	18	6.2	-	-	5	7.7	-	-	13	5.8	-	-	-	-	0.589
IPPV given at birth	110	37.8	-	-	24	36.9	-	-	86	38.1	-	-	0.953	0.538–1.686	0.868
Ventilation	27	9.3	-	-	4	6.2	-	-	23	10.2	-	-	0.579	0.193–1.738	0.330
Number of days in ventilation	-	-	4	2–5	-	-	4	2–4	-	-	2	2–5	1.040	0.667–1.621	0.981
NICU admission	31	10.7	-	-	5	7.7	-	-	26	11.5	-	-	0.614	0.227–1.664	0.338
Duration of NICU admission (days)	-	-	6	5–11	-	-	7	5–9	-	-	6	4–11	1.003	0.782–1.287	0.981
Duration of stay at Central Hospital	-	-	20	10–33	-	-	26	13–35	-	-	15	9–29	0.949	0.884–1.019	0.149
Corrected gestational age when swab was taken	-	-	34	33–36	-	-	34	33–36	-	-	35	33–36	0.937	0.839–1.047	0.251
**Antibiotic exposure prior to rectal swab:**															
Ampicillin and gentamicin	163	56.0	-	-	34	52.3	-	-	129	57.1	-	-	0.870	0.501–1.513	0.623
Piperacillin/tazobactam and amikacin	56	19.2	-	-	12	18.5	-	-	44	19.5	-	-	0.843	0.407–1.744	0.644
Vancomycin	9	3.1	-	-	2	3.1	-	-	7	3.1	-	-	0.993	0.201–4.901	0.993
Meropenem	34	11.7	-	-	4	6.2	-	-	30	13.3	-	-	0.428	0.145–1.264	0.125
Total days of antibiotic exposure prior to rectal swab	-	-	2	0–5	-	-	2	0–3	-	-	3	0–5	0.949	0.884–1.019	0.149
Any surgical intervention	5	1.7	-	-	1	1.5	-	-	4	1.8	-	-	0.867	0.095–7.896	0.899
NEC (medical and surgical)	20	6.9	-	-	4	6.2	-	-	16	7.0	-	-	0.861	0.277–2.670	0.795
Outcome – demised†	11	3.8	-	-	2	3.1	-	-	9	4.0	-	-	0.765	0.161–3.634	0.737

IQR, interquartile range; ELBW, extreme low birth weight; VLBW, very low birth weight; LBW, low birth weight; HIV, human immunodeficiency virus; CPAP, continuous positive airway pressure; IPPV, intermittent positive pressure ventilation; NICU, neonatal intensive care unit; NEC, necrotising enterocolitis; OR, odds ratio; CI, confidence interval.

† , after discharged to home.

### Prevalence of rectal colonisation with carbapenem-resistant Enterobacterales

Overall CRE rectal colonisation prevalence at the time of transfer from the central hospital was 22.3% (65/291; 95% CI: 17.9% – 27.5%) (Figure 1), with colonising species including *Klebsiella pneumoniae* (59/65; 90.8%) and *Serratia marcescens* (6/65; 9.2%). Carbapenem-resistant Enterobacterales colonisation rates were similar in neonates transferred to the regional hospital compared to the district hospital (36/139 [25.9%] vs. 29/152 [19.1%]; *p* = 0.204).

### Factors associated with carbapenem-resistant Enterobacterales rectal colonisation

There were no factors statistically significantly associated with CRE rectal colonisation (Table 1).

### Antibiotic usage

Prior antibiotic exposure was documented in 182/291 (63%) transferred neonates, including ampicillin 166/291 (57%), gentamicin 160/291 (55%), amikacin 55/291 (18.9%), piperacillin-tazobactam 56/291 (19.2%), azithromycin 3/291 (1%), cefotaxime 9/291 (3.1%), vancomycin 9/291 (3.1%), and meropenem 34/291 (11.7%). There was no difference in CRE colonisation rates by antibiotic exposure status; median antibiotic exposure duration was 2 days for the CRE colonised population and 3 days for the non-CRE colonised group.

### Duration of carbapenem-resistant Enterobacterales colonisation

Of the 65/291 neonates who screened positive for CRE colonisation at hospital transfer, a subsequent CRE rectal colonisation screening specimen was collected with readmissions in 35/65 (53.8%) neonates. Nine out of 35 (26%) remained CRE-colonised. The longest duration of colonisation was 6 months in two patients who were readmitted.

### Hospital and long-term neonatal outcomes

Following identification of positive CRE colonisation status, no neonate developed CRE culture-confirmed bloodstream infection. Because of the unavailability of colistin at both district and regional hospitals, none of the neonates were treated with colistin if there were clinical concerns of a possible hospital-acquired infection, and none of the neonates were transferred back to the central hospital for colistin therapy. There were no in-hospital mortalities prior to discharge. Of the 291 neonates, 11 (3.8%) died in the year following discharge from the regional or district hospital. This includes all deaths that occurred in patients readmitted to hospital post-neonatal discharge, deaths that occurred in the emergency department and patients who were dead on arrival to the hospital, but excludes deaths not registered at the hospitals. Post-discharge mortality rates were similar in the CRE-colonised versus the non-colonised neonates (2/65 [3.1%] vs. 9/226 [4.0%]; *p* = 0.737) (Table 2). Of the 7 infants, 5 whose cause of death was known, infectious diseases (pneumonia and gastroenteritis) predominated.

**TABLE 2:** Death review performed at 12 months post-initial rectal carbapenem-resistant Enterobacterales swab.

Patient	Age at death	Gestational age at birth in weeks	Risk factors	Duration between discharge and death	Death as in patient or in emergency centre (EC)	Cause of death	Carbapenem-resistant Enterobacterales-colonised initial swab
1	2 months	29	ELBW, twin	12 days	EC	Unknown	Yes
2	8 months	35	IUGR, VLBW, exposed to HIV	7 months	EC	Unknown	Yes
3	11 months	37	Foetal alcoholic syndrome, IUGR, LBW, cyanotic congenital heart disease	6 months	EC	Unknown	No
4	4 months	36	Congenital hydrocephalus	2 months	In patient	Acute gastroenteritis	No
			Social issues in foster care			Severe acute malnutrition	
5	10 months	34	Cyanotic congenital heart disease, LBW, social issues	8 months	In patient	Pneumonia	No
6	1 month	31	VLBW, mom Group B Strep carrier	13 days	EC	Pneumonia	No
7	19 days	39	Severe hypoxic ischaemic encephalopathy, seizures	N/A	In patient	Birth asphyxia encephalopathy	No
8	2 months	31	Teenage mom (15) LBW, respiratory distress syndrome	25 days	EC	Unknown	No
					DOA		
9	3 months	32	VLBW, respiratory distress syndrome, twin	42 days	EC	Pneumonia	No
10	7 months	32	VLBW, respiratory distress syndrome, dysmorphic	5 months	EC	Viral lower respiratory tract infection, rhinovirus isolated	No
11	3 months	30	VLBW, respiratory distress syndrome	25 days	In patient	ALTE with severe hypoxic ischaemic brain injury	No

ELBW, extreme low birth weight; IUGR, intra uterine growth retardation; VLBW, very low birth weight; HIV, human immunodeficiency virus, LBW, low birth weight; EC, emergency centre; DOA, dead on arrival; ALTE, acute life threatening even.

## Discussion

In this study, the overall CRE rectal colonisation prevalence at transfer from the central hospital was 22.3% (65/291), with colonising species including *K. pneumoniae* (59/65, 90.8%) and *S. marcescens* (6/65; 9.2%). No risk factors were significantly associated with CRE colonisation. No CRE-colonised neonate subsequently developed CRE infection. Post-discharge mortality rates were similar in the CRE-colonised versus the non-colonised neonates.

The prevalence of CRE colonisation in this cohort of preterm South African neonates was high (22%) compared to rates published from a Brazilian NICU (2.6%),^20^ India (5%),^11^ and Cambodia (7.5%)^31^ but much lower than that reported from neonatal units in Kenya and Nigeria (62%)^32^ and the Philippines (55%).^19^ The observed prevalence of CRE colonisation in neonates transferred from the central hospital is likely an underestimate, as it excluded neonates already known to be CRE infected/colonised and all neonates too unstable or too small for transfer. Other elements that could contribute to false-negative results include the sampling and culture techniques, intermittent shedding, and the level of bacterial load.^24,25,26^

In previous studies, preterm neonates were at higher risk of developing CRE colonisation than term neonates, presumably owing to underdeveloped immunity, impaired barrier function, greater use of invasive devices, and prolonged hospitalisation.^11,12^ Despite not achieving statistical significance, very low birth weight (VLBW) neonates (as compared to normal birth weight infants > 2500 g) were 2.3 times more likely to develop CRE colonisation in this cohort.

There were no risk factors statistically significant associated with CRE rectal colonisation. This could be attributed to the limited size of the study population. The participants included a group of stable neonates who were ready to be transferred for weight gain and feeding to district and regional hospitals, which excluded many neonates requiring tertiary care, interventions and neonates known to be CRE colonised or infected. In addition, the study comprised 95% premature infants and prematurity may be a statistically significant factor when compared to a normal neonatal population.

Factors associated with CRE rectal colonisation identified in previous studies include prior carbapenem and vancomycin usage, resuscitation, invasive ventilation, surgery, prematurity, extremely low birth weight, non-exclusive breastfeeding, use of nasogastric tube feeding, and prolonged duration of hospitalisation and stay at a tertiary hospital.^10,11,13,20,22,32,33^

Potential limitations associated with CRE screening were that both mom and neonate were not allowed to be admitted to the KMC unit at the regional hospital, leading to reduced opportunities for bonding and potentially longer hospital stay. Other potential negative effects experienced by mothers of CRE-colonised neonates include emotional stress following prolonged isolation.

Reassuringly we found that no neonates were readmitted with culture-proven CRE infection and that there was no difference in post-discharge mortality rates among CRE-colonised versus non-colonised infants. The mortality rates are lower than a recent study conducted in Cape Town by Kedisaletse Moloto et al., which reported a mortality rate of 6.4% among colonised patients, although this study did not focus specifically on neonates.^34^

Post-discharge mortality occurred in 3% – 4% of the infants with infectious causes predominating in the first year after discharge.

Limitations of this study include the retrospective study design, which precluded collection of accurate data on central line use and duration and receipt of total parenteral nutrition.

## Conclusion

Rectal colonisation with CRE was common in preterm neonates being transferred for step-down care. There were no apparent factors associated with CRE rectal colonisation and no increase in CRE infection or mortality post-discharge for CRE-colonised compared to non-colonised infants. Therefore, even though the prevalence of rectal colonisation was high, we recommend against routine CRE screening in LMICs; however, we strongly recommend screening during an outbreak. The foundation for preventing and controlling the colonisation and infection of CRE lies in implementing stringent infection IPC measures and effective antibiotic stewardship. This aims to reduce the overuse of carbapenems and minimise the duration of their use.

Further research is needed locally and internationally to understand the true impact of CRE colonisation and to guide CRE screening policies and management of colonised infants to prevent CRE outbreaks. In addition, multicentre, prospective studies are needed to establish the effect of interventions such as probiotic therapy and KMC on CRE colonisation rates.
